# Fatal Tension Hemothorax Combined With Exanguination: A Rare Complication of Neurofibromatosis

**DOI:** 10.5811/cpcem.2019.7.43432

**Published:** 2019-09-18

**Authors:** Roz Bidad, Caroline Hall, Eike Blohm

**Affiliations:** University of Vermont Medical Center, Department of Emergency Medicine, Burlington, Vermont

## Abstract

Neurofibromatosis (NF) is a common autosomal dominant disorder that can be subdivided into type 1, type 2, and schwannomatosis. Patients with NF1 typically develop café-au-lait spots, scoliosis, and benign neurofibromas. In addition, NF1 predisposes to vascular complications including stenosis, arterial ectasia, and aneurysms. Here, we report the case of an otherwise healthy 32-year-old man who developed a fatal tension hemothorax due to vertebral artery aneurysm rupture. Based on the available literature, we discuss the presentation, workup, and available therapeutic approaches to this complication of neurofibromatosis.

## INTRODUCTION

Approximately 100,000 individuals in the United States have neurofibromatosis (NF), an autosomal dominant disorder categorized as type 1 (NF1), type 2 (NF2), or schwannomatosis (SWN). The most common form, NF1, affects about 1 in 3,000 people in the U.S. and manifests by the age of 10; it is due to spontaneous mutation in almost 50% of cases.^1^ Symptoms of NF1 present as skin abnormalities including café-au-lait spots, Lisch nodules and freckling in the axilla and inguinal regions. Between 0.4–6.4% of patients with NF1 develop vascular abnormalities, including arterial ectasia, stenosis, and aneurysms.^1^

The vascular malformations as well as the friable tissues of the innumerable tumors predispose to spontaneous hemorrhage.^2^ Neurofibromata can erode into the vascular system, or bleeding can occur from the vascular supply of the tumors. 3 Due to the abundance of vessels in the thoracic cavity, patients with NF1 are at risk of developing a spontaneous hemothorax. Bleeding into the thoracic cavity may present with chest pain or shortness of breath developed from reduced tidal volume.^4^ We report a case of a fatal tension hemothorax due to extrathoracic aneurysmal rupture in a patient with NF1.

## CASE REPORT

A 32-year-old man with pertinent past medical history of NF1 and remote surgical spinal fusion (C3-T11, performed 11 years prior) presented to the emergency department (ED) with an acute exacerbation of his chronic neck pain without focal neurological complaints. The exacerbation occurred the previous day, and the patient unsuccessfully attempted to control his symptoms with prescribed opioid analgesics. He had experienced multiple syncopal events with movement, which he attributed to the intensity of his pain.

Initial vital signs showed sinus tachycardia at 125 beats per minute and tachypnea at 35 breaths per minute. The patient was afebrile (36.3 ° Celsius), normotensive (121/72 millimeters of mercury (mmHg), and oxygenated well (pulse oximetry oxygen saturation 97%) on room air. Physical exam showed a well-developed but ill-appearing young man. He was pale with reduced breath sounds over the right hemithorax. On cardiovascular exam, jugular venous distention, murmur, or peripheral edema were absent. There was no external evidence of trauma. A rectal exam was negative for blood.

**Image 1:**
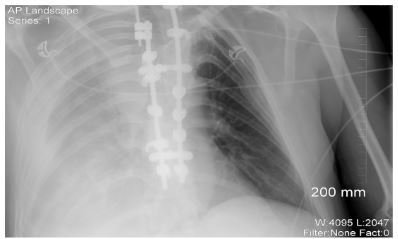
Portable chest radiograph showing significant right-sided extrapleural fluid.

Initial laboratory evaluation showed significant leukocytosis at 35.8 k/mm^3^ (thousand cells per cubic millimeter) (4.0–10.4 k/mm^3^), hemoglobin 9.9 grams per deciliter (g/dL) (13.8–17.3 g/dL), lactate 9.2 millimoles per liter (mmol/L) (<2.1 mmol/L), creatinine 1.8 milligrams (mg) per deciliter (mg/dL) (0.66–1.2 5 mg/dL), and venous pH of 7.26 (7.35–7.45) with partial pressure of carbon dioxide of 34 mmHg (35–45 mmHg), and bicarbonate of 16 mmol/L (23–27 mmol/L). No prior recent laboratory values were available for comparison. Point-of-care ultrasonography was negative for pneumothorax, pericardial effusion, right heart strain, peritoneal fluid, and abdominal aortic aneurysm. Right extrapleural fluid and collapsible inferior vena cava were noted. A portable supine anterior-posterior chest radiograph (Image 1) again showed right-sided extrapleural fluid.

The patient’s condition deteriorated approximately 15 minutes after arrival to the ED. He became unresponsive and shortly thereafter suffered a ventricular fibrillation cardiac arrest. Cardiopulmonary resuscitation (CPR) was initiated, and the patient had return of spontaneous circulation after eight minutes of CPR with two attempts of asynchronous cardioversion and 1 mg intravenous (IV) epinephrine. During chest compression, new right-sided neck swelling developed. Point-of-care ultrasonography showed free fluid between the soft tissue layers. The patient was intubated without complications. Several minutes later, he developed pulseless electrical activity (PEA) and CPR was resumed.

A transesophageal echocardiogram performed during CPR pulse-checks showed coordinated myocardial contraction too weak to produce palpable pulses (pseudo-PEA). A needle aspiration of the right thoracic cavity revealed frank blood, coupled with the collapsible inferior vena cava, pseudo-PEA, and radiograph findings the diagnosis of hemothorax and subsequent cardiac arrest due to internal exsanguination was made. The patient received four units of packed red blood cells and had return of palpable pulses. The decision was made to defer placement of a chest tube as an autologous blood recovery system was not immediately available, the patient oxygenated well, and thoracostomy would have relieved any hydrostatic pressure on the source of hemorrhage, thus hastening the patient’s exsanguination. The patient remained hemodynamically stable for 15 minutes, and the decision was made to obtain imaging to plan for either endovascular or surgical hemorrhage control.

**CPC-EM Capsule**

What do we already know about this clinical entity?

*Patients with neurofibromatosis are prone to the development of a spontaneous hemothorax due to intrathoracic neurofibromata as well as intrathoracic aneurysms*.

What makes this presentation of disease reportable?

*We report the first case of an extrathoracic vertebral artery aneurysm rupture that resulted in the development of a tension hemothorax*.

What is the major learning point?

*Hemorrhage control may be challenging. Thoracostomy predisposes to exsanguination, while massive transfusion protocols can create tension physiology*.

How might this improve emergency medicine practice?

*Incidentally-found, unruptured aneurysms in patients with neurofibromatosis should undergo urgent evaluation, as rupture is often fatal*.

**Image 2:**
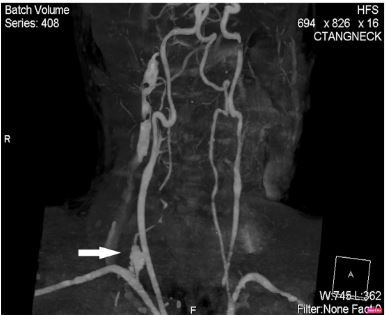
Computed tomography angiogram showing an aneurysm (arrow) at the right vertebral artery origin with poor opacification of the remainder of the vessel.

Computed tomography of the neck and chest detected a ruptured, right-sided vertebral artery pseudoaneurysm at the origin from the subclavian artery with active extravasation (Image 2). The local hematoma had dissected through the soft tissues of the neck into the right hemithorax. Interventional radiology and vascular surgery were consulted to assist with hemorrhage control, and the patient was transferred to the intensive care unit. Massive transfusion protocol was initiated but the patient became progressively hypotensive despite vasopressor therapy. During preparation for chest tube placement, the patient again went into PEA arrest and CPR was initiated. During resuscitation, a right-sided chest tube was placed emergently and clamped after two liters of blood had been evacuated. After tube thoracostomy, the patient had a change in rhythm from bradycardic PEA to sinus tachycardia with detectable pulses shortly thereafter.

After tube thoracostomy, the patient emergently proceeded to the operating suite. En route, he again suffered a PEA cardiac arrest due to exsanguination after evacuation of the right hemothorax. Despite ongoing resuscitation attempts, the patient expired in the operating room.

## DISCUSSION

We report the first case of a tension-type hemothorax due to NF. Given the acuity of the patient, no imaging was obtained to radiographically verify the interim development of a tension hemothorax. The immediate improvement with thoracic decompression by tube thoracostomy supports the diagnosis, as does the preceding massive transfusion protocol which likely added the volume required to convert a hemothorax to a tension hemothorax. It is unlikely that a hypovolemic arrest would have occurred immediately after massive transfusion protocol and would have led to return of spontaneous circulation with CPR and chest tube placement alone.

Hemothoraces have been reported as a rare complication of this disease, typically due to hemorrhage from neurofibromata invasion of thoracic vessels or direct bleeding from the tumors.^4^,^5^ Spontaneous hemothoraces due to aneurysmal rupture have been reported in the literature.^6^–^9^ An overview of the cases with extracranial vertebral artery aneurysms in patients with NF1, their presenting complaints, interventions, and outcome are provided in Table 1.

Primary vascular pathology includes arterial ectasia, stenosis, aneurysm, or fibromuscular dysplasia.^10^ Percutaneous coil embolization and stent-graft placement has been successfully employed,^11^ although surgical management is often required.^12^ The aorta and renal arteries are the most common vessels affected by vascular pathology. A review of the literature showed 20 reports of extracranial vertebral artery aneurysms in patients with NF1.^13^,^14^ Radiculopathy or neck pain appear to be manifestations of unruptured aneurysms, whereas rupture can produce a neck mass with airway compromise or hemothorax.^14^

Due to the high risk of a fatal outcome with rupture, early diagnosis and elective repair prior to rupture is paramount. Vascular abnormalities of any type occur in up to 6.4% of patients^14^; thus, focal pains and paresthesias should be thoroughly evaluated in patients with NF1, with a low threshold for vascular imaging. Once rupture occurs, patients require immediate volume resuscitation and endovascular or surgical intervention.

## CONCLUSION

Tension hemothorax due to vertebral artery aneurysm is a rare complication of NF1. Patients with NF1 who present with neck pain, back pain, radiculopathy, cervical hematoma, or hemothorax should be suspected of having vertebral or intercostal artery aneurysm. Unruptured aneurysms should be urgently addressed with endovascular therapy and followed to prevent re-bleeding. When there is hemodynamic instability, early surgical intervention should be considered to prevent tension physiology from developing. There may need to be routine screening for patients with NF1 for vascular malformations of the cranial arteries. In our patient, differentiating between chronic and acute neck pain potentially delayed diagnosis of vertebral aneurysmal rupture causing the massive hemothorax.

## Figures and Tables

**Table:** Demographics, location, presenting symptoms, interventions, and outcomes of patient with neurofibromatosis type 1 and extracranial vertebral artery aneurysms.

Author (year)	Age (years)/sex	Side	Spine level	Signs and symptoms	Ruptured or unruptured	Treatment	Outcome
Schubiger and Yasargil (1978)	50/Male	Left	C2–C6	Radiculopathy	Unruptured	Surgery	Good
Pentecost et al. (1981)	1/Female	Left	T1	Limited neck movement, arm weakness	Unruptured	Observation	Disabled
Detwiler et al. (1987)	52/Female	Left	C2	Neck mass, neck pain, bruits	Unruptured	Endovascular balloon	Good
Negoro et al. (1990)	43/Female	Left	C1	Neck pain, cervical hematoma	Ruptured	Endovascular balloon	Good
Muhonen et al. (1991)	52/Female	Left	C2	Neck mass, neck pain, arm weakness	Unruptured	Endovascular balloon	Good
Schievink and Piepgras (1991)	43/Female	Left	C7	No symptoms	Unruptured	Observation	Good
Ohkata et al. (1994)	48/Female	Left	C4–C7	Radiculopathy	Unruptured	Surgery	Good
Horsley et al. (1997)	56/Female	Left	C5–C7	Neck pain, arm paresthesias, neck mass	Ruptured	Endovascular coil	Good
Hoffman et al. (1998)	59/Male	Right	C6	No symptoms	Unruptured	Observation	Good
Ushikoshi et al. (1999)	40/Female	Left	C1	Cervical hematoma	Ruptured	Endovascular balloon	Good
Miyazaki et al. (2004)	52/Female	Left	C5–C7	Radiculopathy, hypotension, altered consciousness, hemothorax	Ruptured	Endovascular balloon then surgery	Dead
Arai et al. (2007)	38/Male	Left	Unknown	Chest pain, dizziness, vomiting, hemothorax	Ruptured	None	Dead
Hieda et al. (2007)	36/Female	Left	Ostium of vertebral artery	Back pain, chest pain, dyspnea, hypotension, hemothorax, coma	Ruptured	Endovascular coil	Dead
Hiramatsu et al. (2007)	67/Male	Left	Proximal segment of vertebral artery	Dizziness	Unruptured	Endovascular coil	Good
Pereira et al. (2007)	14/Female	Right	C5–C6	Radiculopathy	Unruptured	Endovascular balloon	Good
Peyre et al. (2007)	18/Female	Right	C5–C6	Radiculopathy	Unruptured	Endovascular coil	Good
Horie et al. (2008)	30/Female	Right	C6–C7	Radiculopathy	Unruptured	Endovascular balloon and coil	Good
Hige et al. (2010)	60/Female	Left	Unknown	Cervical hematoma, respiratory failure	Ruptured	Endovascular coil	Disabled
Morvan et al. (2011)	36/Female	Left	C3–C4	Headache, neck pain, vomiting, subarachnoid hemorrhage	Ruptured	Endovascular coil	Unknown
Author (year)	Age (years)/sex	Side	Spine level	Signs and symptoms	Ruptured or unruptured	Treatment	Outcome
Hiramatsu et al. (2012)	31/Male	Right	C6	Neck pain, cervical hematoma, radiculopathy	Ruptured	Endovascular coil	Good
Gouaillier-Vulcain et al. (2014)	32/Male	Left	C8	Radiculopathy	Unruptured	Surgery and endovascular stent	Good
Uneda et al. (2016)	35/Female	Right	C3–C4	Radiculopathy	Ruptured	Endovascular coil	Good
Present case	32/Male	Right	C7	Neck pain, back pain, syncope	Ruptured	Surgery	Dead

*C*, cervical; *T*, thoracic.
